# Electronic Modulation and Structural Engineering of Carbon-Based Anodes for Low-Temperature Lithium-Ion Batteries: A Review

**DOI:** 10.3390/molecules28052108

**Published:** 2023-02-23

**Authors:** Jiaxun Sun, Lingqian Ye, Xinran Zhao, Peipei Zhang, Jun Yang

**Affiliations:** School of Material Science and Engineering, Jiangsu University of Science and Technology, Zhenjiang 212003, China

**Keywords:** carbon, anode, lithium-ion batteries, electronic modulation, structural engineering, low temperature

## Abstract

Lithium-ion batteries (LIBs) have become the preferred battery system for portable electronic devices and transportation equipment due to their high specific energy, good cycling performance, low self-discharge, and absence of memory effect. However, excessively low ambient temperatures will seriously affect the performance of LIBs, which are almost incapable of discharging at −40~−60 °C. There are many factors affecting the low-temperature performance of LIBs, and one of the most important is the electrode material. Therefore, there is an urgent need to develop electrode materials or modify existing materials in order to obtain excellent low-temperature LIB performance. A carbon-based anode is one candidate for use in LIBs. In recent years, it has been found that the diffusion coefficient of lithium ion in graphite anodes decreases more obviously at low temperatures, which is an important factor limiting its low-temperature performance. However, the structure of amorphous carbon materials is complex; they have good ionic diffusion properties, and their grain size, specific surface area, layer spacing, structural defects, surface functional groups, and doping elements may have a greater impact on their low-temperature performance. In this work, the low-temperature performance of LIBs was achieved by modifying the carbon-based material from the perspectives of electronic modulation and structural engineering.

## 1. Introduction

As one of the components of energy strategy, energy storage devices play an important role in promoting the development and modernization of national informatization. Compared with other types of batteries, lithium-ion batteries (LIBs) have the advantages of high energy density, high power density, long cycle life (thousands of cycles), good safety performance, and low cost. These benefits are accompanied by higher specific energy and operating voltage, longer charge–discharge life, a lower self-discharge rate, reduced environmental pollution, and the absence of memory effects during use [1,2]. Therefore, in a short time, LIBs have begun to dominate the field of energy storage, with high development potential, and are widely used in portable electronic devices such as mobile phones, tablets, and laptops, and in important fields such as transportation and aerospace [3,4]. LIBs also meet the requirements for use in electric vehicles, such as long driving ranges, high current charging, and safety [5,6,7]. Eduardo et al. established the capacity attenuation model of NMC/C batteries in order to study the aging condition of batteries at the State-of-Charge (SOC) level and the current curve used by electric vehicles [8]. This model has a wide range of potential applications in the evaluation of battery usage and optimal charging scheduling for electric vehicles, and in the design of energy management strategies for plug-in hybrid electric vehicles. Park et al. conducted a study to identify small satellite battery systems to ensure the reliability of BMS and LIBs and provide credibility for the use of LIBs in space applications [9]. In addition, Depetro et al. observed that LIBs were likely to significantly improve the capability of conventional submarines [10].

**Figure 1:**
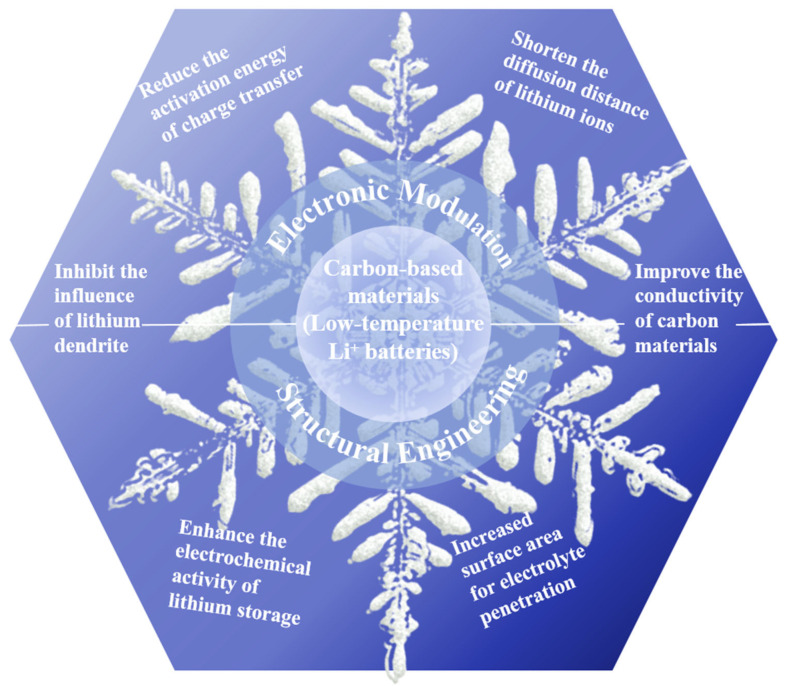
A schematic illustration of key obstacles and strategies of carbon-based materials for low-temperature LIBs.

The operating and charging temperatures of conventional LIBs are between −20 °C and 60 °C and 0 °C and 60 °C, respectively. However, below 0 °C, the performance of LIBs will generally decline, and the energy and power density of LIBs using commercial electrolytes will decrease sharply when the temperature falls below −20 °C [11,12]. A number of factors, such as a decline in the conductivity of the electrolyte ions [13], an increase in the impedance value of the solid electrolyte interface (SEI) membrane [14,15], and a reduction in the rate of lithium-ion diffusion within the positive and negative electrode materials, affect the performance of LIBs at low temperatures [16,17] and result in reduced discharge capacity. The decrease in the discharge platform and discharge capacity caused by low temperature will shorten the battery lives of electrical equipment or cause them to shut down in practical applications. Under low-temperature conditions, high-rate charging will cause lithium metal precipitation, which triggers a lithium metal and electrolyte reaction involving excessive consumption of lithium and electrolyte, and results in irreversible capacity loss [18,19]. In addition, the deposition on the electrode surface increases the impedance, which further hinders the embedding of the lithium ion in the anode, and the precipitated lithium metal may form dendrites, which may puncture the diaphragm and cause a short circuit and thermal runaway in the battery. In practical application, it will shorten the service life of electrical equipment and can even create safety risks [20]. This severely limits their use in scientific exploration and areas of military strategic importance, such as in alpine and polar regions, and in space. Therefore, achieving outstanding low-temperature performance is a critical issue for resolution in the development of LIBs. A large number of research groups have reviewed extensive research advances and possess an in-depth understanding of the important variables contributing to the poor low-temperature performance of LIBs [21,22]. They sorted out the distinctive challenges on the anodes, electrolytes, cathodes, electrolyte-electrodes interphases, and so on, which highlight the importance of low-temperature LIBs [23,24,25]. In particular, many discussions have been made on the nanoadjustment of carbon-based materials for LIBs-related applications [26,27,28]. This study aims to highlight the fundamental issues relevant to the topic, including the electronic modulation and structural engineering of carbon-based anodes, as a complement to the recently published reviews of low-temperature LIBs (Figure 1). Finally, we offer our opinion on the future development of LIBs for use at low temperatures.

## 2. LIBs at Low Temperatures

### 2.1. Limitations of LIBs at Low Temperatures

The low-temperature performance of LIBs is influenced by a variety of factors. These include external factors such the packaging of the LIBs, the external temperature, and the charging cut-off voltage. The type of electrolyte, positive and negative electrode composition, and mode separation density of LIBs will all have a significant impact on their low-temperature performance because, from an internal perspective, LIBs are primarily composed of an electrolyte, a positive and negative electrode, and a separator. The performance of batteries at low temperatures is primarily influenced by the low conductivity and diffusion rate of lithium ions in the electrolyte and electrode, as well as the high impedance of the SEI film. At low temperatures, LIBs are often restricted regarding these three aspects and there is a need for a new battery system for operation at low temperatures. We will discuss these aspects below.

#### 2.1.1. Electrolyte

**Figure 2:**
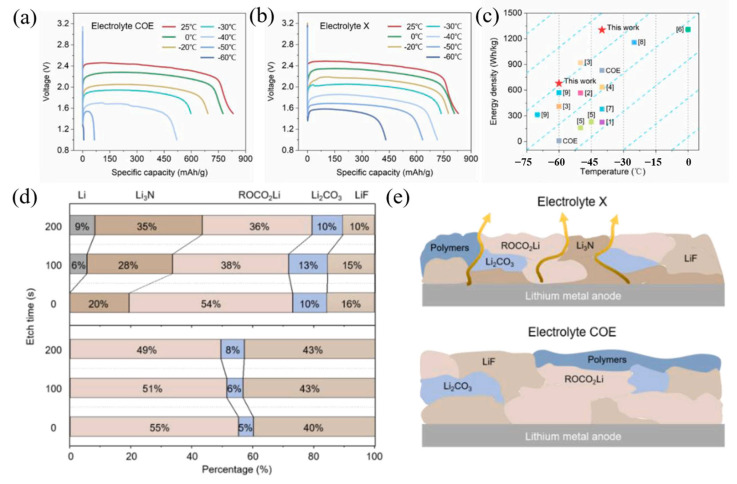
Discharge curves of Li/CFx primary cells using the (**a**) electrolyte COE and (**b**) electrolyte X at different temperatures (0.1 C). (**c**) The comparison with the same type of research on the energy densities of Li/CFx cells available at different temperatures. (**d**) The relative contents of lithium-containing components and (**e**) schematic diagrams of the compositions of SEI formed in the two electrolytes. (**a**–**e**) Reproduced with permission from [31].

The first limitation is the electrolyte as an ion-conduction medium. Due to the transmission of solvated lithium ions in the liquid electrolyte, low temperatures will directly lead to an increase in the viscosity of the electrolyte, which reduces the migration rate and conductivity of the ions. In addition, the incompatibility of the electrolyte and electrode results in an increase in the resistance of the solid–liquid interface, which adversely impacts the cycle stability and cell density of the LIB [29]. Many scholars have proposed improvements to resolve this problem [30]. A lithium/fluorine fossil ink (Li/CF_x_) battery with an optimized electrolyte exhibits significantly improved low-temperature performance, according to research by Li et al. [31]. This is because the optimized electrolyte formula fully exploits the synergistic benefits of many components. As shown in Figure 2, its capacity at −60 °C can be greater than 52% of that at normal temperatures, and at −60 °C it can deliver 679.4 Wh kg^−1^ of energy density (based on cathode active material), which provides theoretical and experimental design guidelines for logical low-temperature electrolyte design. TiO_2_ has good potential as an anode for energy storage [32]. Zhang et al. designed an optimized TiO_2_(B) negative electrolyte formula for LiBF_4_-based LIBs, in which the optimized ratio electrolyte TiO_2_(B) anode demonstrated a reversible capacity of 240 mAh g^−1^ at −20 °C (0.1 A g^−1^) [33]. Based on a molecular dynamics simulation, the separation of lithium ions and solvents in the optimized formula helped to increase electrochemical performance. TiO_2_(B) exhibited outstanding electrochemical performance in this tailored electrolyte at low temperatures up to −30 °C. In their work, Li et al. constructed a low-impedance anode interphase by applying the electrolyte additive dimethyl sulfate (DMS), which changes the chemical properties of the SEI [34]. The surface plays an important role. It has a weak binding and stable structure with lithium ions, and thus reduces interface impedance and inhibits the poor reduction and decomposition of carbonate solvent and LiPF, resulting in a significant improvement in the low-temperature performance of LIBs.

#### 2.1.2. SEI Film

Secondly, suitable SEI film can also significantly improve battery performance at low temperatures. X. Liu et al. presented a novel strategy to create a more stable interface between the electrolyte and electrode by constructing a sulfuric acid-containing SEI layer on top of a silicon-based anode to achieve a SiO/C anode with high performance at low temperatures [35]. The SiO/C composite anode was modified with an electrolyte containing organic–inorganic hybrid components during initial discharge. The modified layer changes the decomposition path and dynamics of lithium ions at the interface, thus promoting its dissolution behavior at low temperatures and providing a new perspective for the development of advanced SiO/C anodes and low-temperature LIBs. To create a conductive SEI made of a thick and protective inner inorganic layer on the surface of the graphite, Shi et al. used fluorosulfonyl isocyanate (FI) as a new type of SEI film-forming electrolyte additive for graphite anode. This effectively prevented the growth of an external organic layer and significantly decreased the resistance of the graphite/electrolyte interface [36]. The lithium/graphite cells with 2 wt% FI demonstrated exceptional rate performance at both ambient (20 °C) and low temperatures (0 °C and −20 °C), compared with the lithium/graphite cells employing the reference liquid electrolyte (LP30).

#### 2.1.3. Development of Other Low-Temperature Battery Systems

In order to resolve the performance defects of LIBs at low temperatures, new battery systems such as all-solid-state LIBs, ion capacitors, aqueous LIBs, and sodium-ion batteries (SIBs) can also be used, and the performance of these new battery systems can be improved under low-temperature conditions [37,38,39,40,41]. For all-solid-state LIBs in low-temperature conditions, developing the high conductivity of the solid electrolyte is the key [42]. Thenuwara investigated the adjustment of the solid electrolyte (SEI) structure using two kinds of electrolyte solvent (ring carbonate and ether), causing a sharp increase in the Coulombic efficiency of the lithium metal anode at a low temperature (60 °C) [43]. The results showed that the rechargeable lithium metal battery could achieve energy storage across a wide temperature range. Water-based energy storage has the advantages of ease of operation and low cost, as well as being environmentally friendly [44]. For water-based LIBs, Tron et al. expanded the temperature range of the water-based electrolyte by using ethylene glycol, improved the cycling performance of the electrolyte, reduced the polarization of the electrode reaction, and controlled the improvement of the lithium-ion (de)intercalation process in the nano-LiFePO_4_ material by adjusting the antifreeze content, thus improving performance at subzero temperatures [45]. For SIBs, it is vital to improve the limitation of the slow diffusion kinetics of sodium ions in the active material and the interface. Zhou et al. adopted the strategy of inducing anion ligands between two-dimensional NbSSe nanosheets to consolidate the interlayer band gap and optimize the electronic structure, so that two-dimensional NbSSe nanosheets provided acceptable rates, lifetime electrochemical performance, and had a high reversible capacity of 136 mAh g^−1^ at 0 °C [46]. The outcomes demonstrated that the two-dimensional NbSSe nanoparticle is crucial in enhancing the low-temperature performance of SIBs. To improve the low-temperature tolerance of water-based SIBs, Cheng et al. created SiO_2_-Na_2_SO_4_ hydrogel electrolyte as a water-based sodium-ion electrolyte with activated carbon (AC) serving as the anode and carbon-coated NaTi_2_(PO_4_)_3_(NTP@C) serving as the cathode [47]. SiO_2_ and Na_2_SO_4_ have an intermolecular link that makes it difficult for Na_2_SO_4_ to precipitate or grow continuously in the saturated electrolyte at low temperatures. With a reversible capacity of 61.8 mAh g^−1^ (based on NTP@C), a current density of 0.13 A g^−1^ (0.13 mA cm^−2^), and a high degree of cyclic stability, the battery operates at −30 °C. The battery offers a novel route for the creation of water-based SIBs that can operate at low temperatures. Additionally, the energy cycle can alternately charge and discharge the lithium-ion secondary battery, which uses its internal resistance as a heating element to boost its own temperature and expand its ability to discharge. In order to improve battery performance at low temperatures, it is essential to develop an energy cycle operation method and device based on the electrochemical impedance spectroscopy of lithium-ion secondary batteries. The heat produced by internal resistance during the energy cycle operation of lithium-ion secondary batteries at extremely low temperatures increases the battery temperature, demonstrating that the internal temperature of the lithium-ion secondary battery can be raised during energy cycle operation in low-temperature environments.

#### 2.1.4. Electrode

Although numerous studies have been conducted to enhance the electrolyte and SEI film of LIBs and certain advancements have been made in the performance of LIBs at low temperatures, the main factor affecting the low-temperature performance of LIBs is the electrode material. The low diffusion rate and slow desolvation process of Li ions at low temperatures have negative effects on both anode and cathode materials. The negative effect of low temperatures on LIB electrodes can be resolved by selecting or developing electrode materials suitable for low-temperature operation [48]. For this purpose, Xu et al. improved the low-temperature performance of LIBs through the development of an electrode which was made of thin graphite sheets with through-holes and carbon nanotubes. This electrode successfully prevented the restacking of graphite sheets through the carbon nanotubes [49]. Using multiheteroatom doping and the addition of MXene, Pu et al. created lithium titanate (LTO) nanosheets with a large specific surface area and enhanced their electrochemical performance [50]. Using multiheteroatom doping and the addition of MXene, Z. Pu et al. created lithium titanate (LTO) nanosheets with a large specific surface area and enhanced their electrochemical performance (the inherent conductivity is only 10^−9^ S cm^−1^ for LTO), which helps to improve the low-temperature electrochemical performance of LIBs. Jia et al. synthesized nanosized vanadium nitride (VN) coated with carbon fiber at 800–1000 °C by molten salt disproportionation synthesis [51]. After 500 cycles, VN, the anode material for LIBs, had a reversible discharge capacity of 320.1 mAh g^−1^ at 1.0 A g^−1^. The reversible discharge capacity was 669.4 mAh g^−1^ after 230 cycles with no capacity attenuation, which helped to boost the storage capacity of lithium ion and promoted the performance of LIBs at low temperatures. The cathode of Li_1.2_Ni_0.13_Co_0.13_Mn_0.54_O_2_ was prepared by simple AlF_3_ coating via wet chemical processing for low-temperature LIBs, which found in Li_1.2_Ni_0.13_Co_0.13_Mn_0.54_O_2_ covers a nanoscale particle surface layer thickness of the dense AlF_3_ adjacent active material for building the rapid transport of lithium bridges [52]. The discharge capacity and initial Coulombic efficiency were increased, whereas the side interactions between the electrolyte and the active material were diminished. In addition, the sample coated with 2% AlF_3_ showed an obviously superior rate and capacity retention ability at 0 °C, which effectively improved the low-temperature performance of the LIBs.

## 3. Modification of Electrode Materials

The most crucial element that affects the low-temperature electrochemical performance of LIBs is the active material of the electrode. Lithium cobalt oxide, lithium nickel oxide, lithium manganese oxide, and other conventional cathode materials are available. By incorporating the Co element into the transition metal site, Luo et al. were able to control the relationship between the diffusion and temperature of the LiNi_0.5_Mn_1.5_O_4_ cathode (the electrical conductivity falls in the range of 10^−5^ to 10^−7^ S cm^−1^). They discovered that this improved the ability of the lithium ions to diffuse and increased the electronic conductivity of the materials [53]. These findings not only make it possible to enhance the electrochemical performance of cathode materials based on 5 V, but also offer crucial direction for their practical use at low temperatures. Ye et al. used amorphous Zr-3 (PO_4_) surface engineering to achieve stable high-pressure LiCoO_2_ operation (4.6 V) at 25 °C [54]. A high-quality cathode–electrolyte interface phase, which had excellent stability and low interface resistance at low temperatures, was obtained due to the high degree of amorphousness of the surface layer. The experiment proved that surface control engineering is a useful technique for enhancing the performance of LiCoO_2_ at high pressures and low temperatures.

Anode materials include carbon-based anode, lithium titanate anode, and silicon carbon anode [55,56,57]. The sluggish solid-state lithium-ion diffusion in the positive and negative materials, which prevents the rapid release and storage of significant amounts of lithium ions in LIBs, is the primary cause of the rate capacity constraint of LIBs. Anode materials play an important role in determining the capacity and lithium-ion diffusion rate of LIBs.

### 3.1. Carbon-Based Electrodes for LIBs

**Figure 3:**
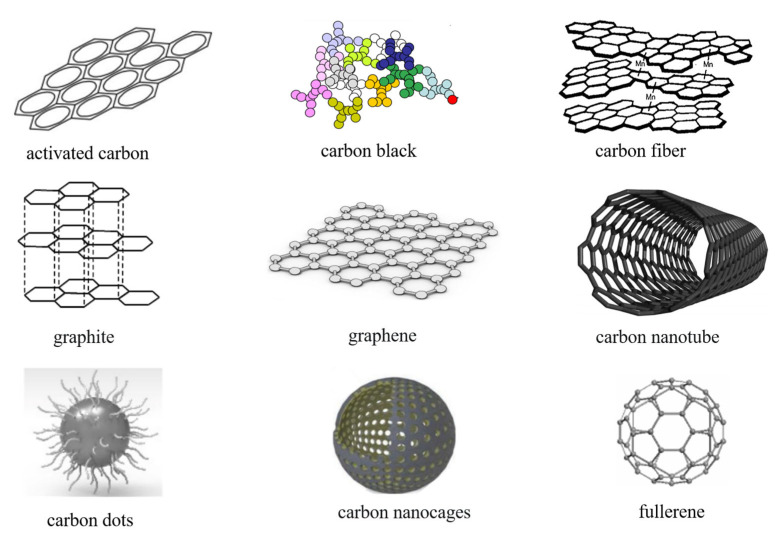
Structures of various carbon-based materials. Reproduced with permission from [61,62,63,64,65,66,67,68,69].

Today, the mainstream anode materials are primarily carbon-based. Carbon-based materials have low resistivity, are low-cost and abundant, and are widely used at room temperature as a power source for various electronic devices and as the main power source for electric vehicles [58,59,60]. Carbon-based materials play an important role in the electrodes of LIBs. As shown in Figure 3, carbon-based materials can be roughly divided into: traditional industrial carbon, including activated carbon [61]; carbon black [62]; new industrial carbon, including carbon fiber and graphite [63,64]; new carbon nanomaterials, including graphene [65]; carbon nanotubes (CNTs) [66]; carbon dots [67]; carbon nanocages [68]; and fullerenes [69].

Activated carbon performs well and is cheap and environmentally friendly. Lithium ions may be readily absorbed by activated carbon when used as LIB electrodes, and the low-temperature treatment of activated carbon results in a material with a high specific surface area of 1728 m^2^ g^−1^ and outstanding specific capacity [70]. This can increase the pore size of the material and enhance the low-temperature electrochemical performance of the LIBs.

The most popular conductive paste for LIBs today is carbon black. It is an amorphous carbon that has a large specific surface area, low density, and strong chemical inertia. Under the scanning electron microscope, it is chain- or grape-shaped, light, and loose, with a small particle size, large specific surface area, poor dispersion, easy aggregation in slurry, and strong oil absorption. However, conductive carbon black cannot form a good conductive network and has relatively poor conductivity (The conductivity of conductive carbon black used in the battery industry is 0.2–0.5 S cm^−1^), high resistance, and easy polarization. Therefore, conductive carbon black is often used together with graphite, CNT, graphene, and other materials in the manufacturing of electrode sheets [71].

High axial strength and modulus, low density, resistance to extremely high temperatures in nonoxidizing environments, good fatigue resistance, specific heat and conductivity between nonmetals and metals, a low coefficient of thermal expansion and anisotropy, good corrosion resistance, good conductivity, and electromagnetic shielding are just a few of the many excellent qualities of carbon fiber. Xu et al. investigated the fiber–matrix adhesion between carbon fibers of different sizes and two different matrix systems using the microbonding test supported by transverse tensile testing, and discovered that the mechanical adhesion of the fiber–matrix interface was lower than that of the commercial nonionic conductive polymer matrix, which is sufficient for LIBs. The matrices in structural batteries must be capable of conducting ions and transferring loads between fibers [72].

The most common anode material in industrial batteries is graphite. At low temperatures, it typically performs poorly in terms of rate. High current rates can also cause lithium plating and dendrite growth on the surface of the graphite, which reduces low-temperature performance and creates safety risks. In addition, the theoretical specific capacity of graphite material is only 372 mAh g^−1^, and with a decrease in temperature, its capacity will also decrease significantly. Its capacity quickly degrades to just 12 mAh g^−1^ at −20 °C. Gunawardhana et al. used chemical vapor deposition (CVD) to uniformly cover graphite with carbon to modify the graphite itself [73]. Different amounts of carbon coating on the surface of natural graphite were used to suppress the amount of lithium deposited at −10 °C and reduce the deposition of lithium on the surface of the graphite. This method provides a uniform carbon coating covering the entire graphite surface, inhibits the unnecessary active points on the graphite, and forms an optimized SEI layer, resulting in better lithium intercalation and deintercalation performance at low temperatures. In addition, it effectively inhibits the deposition of lithium on natural graphite, which is very effective for improving the safety of LIBs at low temperatures. In addition to having potential uses in machinery, energy storage, catalysis, electronic devices, and environmental treatment, graphene has a sheet-like structure and exceptional physical properties, such as a high surface area ratio, high electron mobility, and high thermal conductivity. It can be used as a conductive agent to reduce the amount of conductive agent required (10^6^ S m^−1^). However, the sheet-like structure of graphene also prevents the diffusion of lithium ions, resulting in lower battery multiplier performance. The cellulose/graphene paper developed by Zhang et al. has good mechanical properties and excellent electrochemical performance [74]. Excellent charge–discharge stability after 1600 cycles was achieved using cellulose/graphene paper as the negative electrode in LIBs.

CNTs have seamless cylindrical nanostructures composed of carbon atoms with special chirality, outstanding composite physical properties, high electrical conductivity (up to 10^8^ S m^−1^), and high impact resistance [75]. Compared with traditional graphite-based anodes, CNT-based anodes have a higher reversible lithium-ion capacity and the open structure of CNTs enhances the electrical transmission and capacity of CNT-based LIBs. Furthermore, CNTs can be placed into freestanding electrodes without the use of an adhesive or collector, increasing the specific energy density throughout the entire battery design.

As a relatively new fluorescent carbon-based nanomaterial, carbon dots are characterized by excellent biocompatibility, dimmable luminescence (PL), high quantum yield, and unique electronic and physicochemical properties. In addition, the excellent PL properties of carbon dots have been widely exploited in biological imaging, light-emitting diodes (LED), and other fields [76].

Carbon nanocages are cagelike nanocarbon materials formed by carbon layer curling, and have high a specific surface area, hierarchical distributed pore structure, and excellent electrical conductivity [77]. Nanocages, as opposed to other nanostructures, facilitate the contact between electrode materials and electrolytes and shorten the ion diffusion channel, which creates more space and sufficient electrolyte ions for rapid electrochemical reactions, aiding cycling stability.

Fullerenes are hollow molecules made entirely of carbon, similar in structure to graphite. Fullerenes can be divided into C₂₀, C₆₀, C₇₀, C₇₆, and C₈₀ depending on the total number of carbon atoms. Of these, the highly symmetrical cage structure of C₆₀ gives it high stability, good electrochemical properties, and mechanical support, and it has therefore been applied to improve the chemical properties of LIBs [78].

Owning to its distinct structure and superior intrinsic physical and chemical properties, graphitic carbon nitride (g-C_3_N_4_), a typical polymeric organic semiconductor, has recently gained increasing interest as an electrochemical energy storage material [79]. In contrast to graphitic layers, g-C_3_N_4_ exhibits regular stacking of C_3_N_4_ layers, which are composed of sp^2^ hybrid conjugated C and N atoms. Additionally, g-C_3_N_4_ is a strong substance with great chemical stability because it is not dissolved in alkali, acidic solution, or organic solvents. Moreover, g-C_3_N_4_ is readily synthesized from a variety of nitrogen-rich precursors, including dicyandiamide, urea, melamine, and thiourea, all of which are inexpensive, environmentally friendly, and naturally abundant in the earth. These characteristics make g-C_3_N_4_ an excellent choice for energy storage materials. However, there are notmany uses for pure g-C_3_N_4_ at the moment due to its poor electrical conductivity and small interlayer spacing distance. Some studies have used g-C_3_N_4_ as an electrolyte additive or in combination with other electrode materials to stabilize properties [80,81].

There is an increasing need for LIBs with high energy density, rapid charging, and a wide temperature range. The purpose of the following sections is to enhance the structural alteration and electronic control of carbon-based anode materials for the low-temperature performance of LIBs. As shown in Table 1, carbon materials can be modified to obtain excellent LIB performance at low temperatures.

#### Electronic Modulation of Carbon-Based Anodes for Low-Temperature LIBs

Altering the surface electron configuration of the carbon anode and directly altering the surface of the LIB anode are two common techniques for controlling the electrons on the surface of carbon atoms. These techniques can facilitate the insertion of a layer of lithium and facilitate the dissolution of lithium ions [92]. The N, S, and P doping method can also be effective in improving the energy storage capacity and movement rate of lithium ions, and thus enable LIBs to be used in extreme conditions [93]. Additionally, the low-temperature performance of LIBs can be improved using the same techniques that are employed to enhance the low-temperature performance of SIBs.

**Figure 4:**
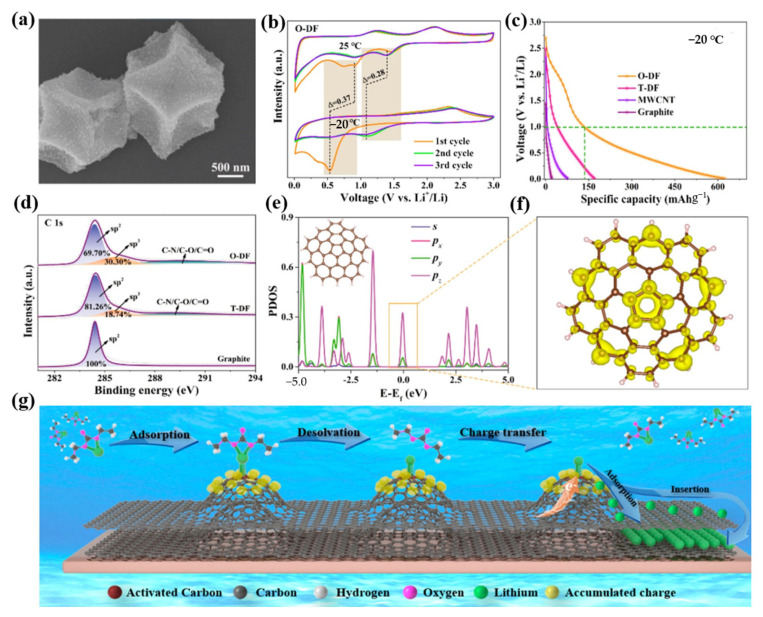
(**a**): SEM images of O-DF. (**b**): CV curves of O-DF for −20 °C at 0.1 mV s^−1^ compared with the corresponding curves for room temperature. (**c**): Galvanostatic discharge curves of O-DF, T-DF, MWCNT, and graphite for −20 °C at 0.1 A g^−1^ after 150 cycles. (**d**): High-resolution XPS C 1s spectra of O-DF, T-DF, and graphite. (**e**): PDOS of C atoms for positive curvature structure. (**f**): Charge density distribution with isosurface values of 0.005 e/Å^3^ corresponding to energy windows of positive curvature carbon in (**e**) near the Fermi level. The yellow represents electron accumulation. (**g**): Accumulated charges originate from the sp^2^/sp^3^-hybridized orbital of the positive curvature structure for low-temperature lithium storage. (**a**–**g**) Reproduced with permission from [94].

The key, according to Yao’s group, is to modify the surface electron configuration of the carbon anode in order to improve coordination between dissolved lithium ions and the adsorption sites, thereby promoting the dissolution of lithium ions and lowering the activation energy of the charge transfer process [94]. They discovered that curved surfaces, particularly those with positive curvature, had a stronger affinity for lithium ions than planes with zero curvature, making it possible to complete the high capacity of carbon anodes in extremely cold environments, as shown in Figure 4a–g. They formed the electronic configuration of the surface through the reaction of the hybrid orbital type generated by the reaction of chemical bonds to bending deformation. The Riemannian and Lobachevsky surfaces are due to the noncoplanar sp^2^ hybridized orbitals which lead to the abundant charge around the Fermi layer of the nonhexagonal defect point and the easy donation of electrons to the corresponding receptors. It is obvious that the accumulated charge on the Riemann surface contributes significantly to lowering the activation energy of the charge transfer during low-temperature lithium-ion storage given the high capacity of O-DF (dodecahedral carbon frame) at low temperatures. They investigated synthetic O-DF and T-DF as LIB anode materials using CR2025 button cells to assess the impact of an atomic-scale Riemann surface and non-Euclidean surface on electrochemical performance at low temperatures. They discovered that O-DF had a much higher discharge-specific capacity than the other materials. It was discovered that the enriched charge of the noncoplanar Riemann surface may efficiently promote the transfer of charge from sp^x^ hybridized carbon to dissolved Li^+^ and boost the capacity for coordination of the intrinsic defect sites. It was demonstrated that in extremely cold settings, Li^+^ can be adsorbing and inserted more readily into noncoplanar Riemann surfaces. Their research showed that the Riemann surface could be used to control the hybrid orbital types of carbon atoms for surface electron modulation, strengthening the coordinated action between dissolved Li^+^ and the adsorption point and inducing noncoplanar sp^x^ hybrid orbitals with unsaturated coordination, in which the local accumulation of charge lowers the energy barrier of the charge transfer during Li^+^ decolonization. This research provides an effective solution to the serious capacity attenuation of the carbon anodes of LIBs in extremely cold environments.

**Figure 5:**
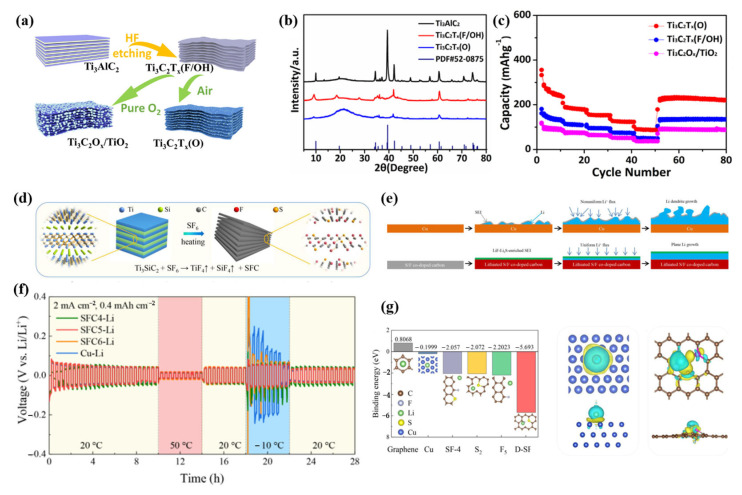
(**a**): Schematic illustration of the fabrication process of pristine MXene Ti_3_C_2_T*_x_*(F/OH), more O functional group substitutions for F in MXene-Ti_3_C_2_T*_x_*(O) and Ti_3_C_2_O*_x_*/TiO_2_. (**b**): XRD patterns of the different samples. (**c**): Rate performances of various materials. (**a**–**c**) Reproduced with permission from [87]. (**d**): Schematic illustration of the synthetic process of the SFC. (**e**): The schematic illustration of the electrochemical lithiation process of Li on a Cu anode or SFC. (**f**): Cycling stability of symmetric cells that incorporated the different electrodes; the temperature was switched repeatedly every 20 cycles between −10 °C and 50 °C. (**g**): Summary of calculated binding energies of Li with Cu, pure graphene, and different doping sites on SFC5. The top view and the side view of the charge density differences of Cu, and d-SF-1 site with one Li atom adsorbed. The lithium, copper, carbon, sulfur, and fluorine atoms are marked as green, blue, brown, yellow, and gray, respectively. (**d**–**g**) Reproduced with permission from [88].

As the temperature decreases, lithium-ion desolubilization energy and electrode polarization increase, resulting in the formation of Li dendritic crystals and the deterioration of battery performance, which seriously affects the low-temperature chemical performance of LIBs [95]. To address this issue, Wang et al. enhanced the low-temperature lithium storage capability of MXene titanium carbide using a surface engineering strategy [87]. They investigated the effects of surface oxygen termination on the effectiveness of lithium storage in MXene materials at low and high temperatures, and they optimized the sintering process to manage the amount of surface oxygen replacement (Figure 5a–c). The surface oxygen-rich Ti_3_C_2_T_x_(O) and Ti_3_C_2_O_x_/TiO_2_ were obtained in air and pure oxygen, respectively. Both of the surface-modified samples maintained the layered structure well, and the surface O-rich Ti_3_C_2_T_x_(O) showed good electrolyte wettability, which promoted the dissolution of lithium ions and facilitated the lithium intercalation layer. At −20 °C and room temperature, Ti_3_C_2_T_x_(O) showed better lithium storage performance, which significantly enhanced the chemical performance of LIBs at low temperatures. One of the main issues affecting the stability and safety of lithium-ion batteries is lithium dendrite growth, which causes the electrode and electrolyte interface to become unstable during the battery cycle, destroys the SEI film created, and continues to consume the electrolyte, resulting in the formation of dead lithium and low Coulombic efficiency. Additionally, the development of lithium dendrites could result in diaphragm puncture, internal short-bonding of the LIBs, thermal runaway, and explosion due to combustion. Therefore, in order to decrease the local current density caused by lithium dendrites and enhance the low-temperature electrochemical performance of LIBs, it is necessary to design a high specific surface area anode. By etching Ti_3_SiC_2_ in an environment containing SF_6_, J. Wang et al. successfully created accordion-like S/F codoped carbon to address this issue. Their lithium–SFC composite electrode displayed dendrite suppression and a minimal voltage lag, as illustrated in Figure 5d [88]. Over a wide temperature range (−10 to 50 °C), the SFC–Li anode and LFP cathode exhibited improved cycle stability and rate capability, as well as small voltage polarization (Figure 5e,f). According to DFT simulations and experimental measurements, the increased electrochemical performance is primarily attributable to the uniform nucleation of lithium and the efficient control of stable SEI layers (Figure 5g). This work represents a new approach to the development of low-temperature, high-energy-density, rechargeable lithium metal batteries.

Additionally, N doping can enhance the electrochemical performance of LIBs at low temperatures while enhancing the electrical conductivity of carbon materials. In this context, Zhang et al. created a three-dimensional N-doped porous carbon framework by sintering N-doped carbon dots (CDots) at 800 °C. [96]. The N-PCFs-based LIBs had a high capacities, as well as higher rate abilities and cycle stabilities, due to their wide surface areas and high N doping concentrations. After one thousand cycles, the laboratory performance reached 840 mAh g^−1^. The findings demonstrate that carbon dots-derived N-PCFs make an excellent anode material for LIBs with good reversibility and extremely stable cycling performance. Using electrospinning and a post-thermal reduction process, L. Lu et al. created a hybrid carbon nanofiber (CNF) nanomaterial with MnO-Sn cubes embedded in nitrogen-doped CNF (MnO-Sn@CNF), which functioned well as an anode for LIBs and had a stable core–shell structure consisting of a three-dimensional conducting network [97]. Additionally, the mesoporous surface of carbon fiber can decrease the lithium-ion diffusion distance and encourage the admixture of lithium ions with active sites. The physical structure of the electrode material is more stable due to the heterogeneous structure created by MnO and Sn within the carbon fiber. The material design technique offers a reference plan for the creation of a high-performance, low-temperature negative electrode for LIBs. Li et al. designed and synthesized a porous N, S-doped carbon framework, in which Mo_2_C particles were embedded as an interlayer material to block polysilicate shuttling [90]. As a catalytic medium, this material is used for lithium–electric conversion. It has an extremely fast wetting ability for electrolytes and a high lithium-ion conductivity. Moreover, it is very effective in inhibiting the shuttle of polymers and promoting the reuse of LiPSs adsorbed at the same time. The battery made from this material had a good rate capacity and long cycle stability, and provides a valuable reference for expanding the applications of Li-S batteries in a wide temperature range.

**Figure 6:**
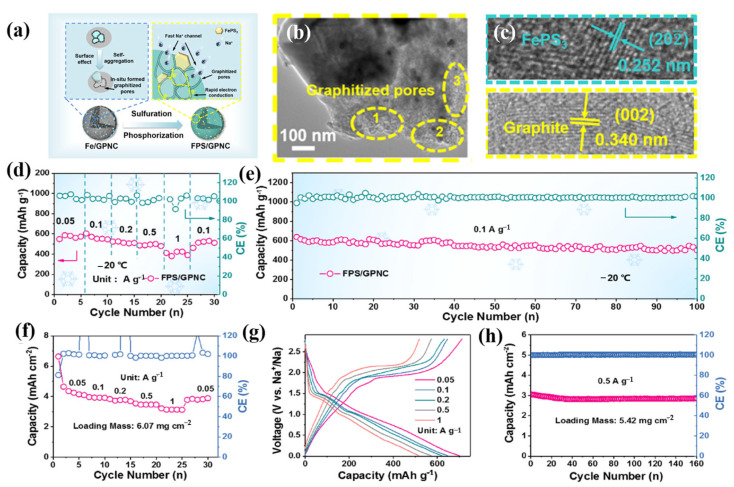
(**a**): Synthesis diagram of the FPS/GPNC composite. (**b**,**c**): HRTEM images of different magnifications, including the local amplification region of graphitized pores and lattices fringes. (**d**): Rate performance of the FPS/GPNC electrode at −20 °C. (**e**): Long-cycling stability of the FPS/GPNC electrode at −20 °C. (**f**): Rate performance of the FPS/GPNC electrode with the loading mass of 6.07 mg cm^−2^. (**g**): Typical voltage profiles at different rates with the loading mass of 6.07 mg cm^−2^. (**h**): Long-cycling performance of the FPS/GPNC electrode with the loading mass of 5.42 mg cm^−2^. (**a**–**h**) Reproduced with permission from [99].

The technique for enhancing the low-temperature performance of SIBs through electronic modification of the negative electrode is also beneficial for enhancing the low-temperature performance of LIBs. In order to improve the low-temperature performance of sodium-ion batteries, Zhou et al. prepared expanded graphite by inserting a small amount of amorphous red phosphorus through oxidation, reduction, and gas deposition processes, which demonstrated good electrochemical performance. At room temperature, it displayed a high reversible specific capacity of 418 mAh g^−1^. In practice, batteries can still be used in extremely cold temperatures because a specific capacity of more than 50 mAh g^−1^ is still accessible at the temperature limit (−50 °C). By the in situ pyrolysis and selenization of PAN@ZIF-8 nanofibers ultrafine ZnSe, X. Wan et al. encapsulated MOF-derived superfine ZnSe nanoparticles in nitrogen-doped porous carbon nanofiber composites (ZnSe@NCNFs) [98]. ZnSe@NCNF anodes showed excellent sodium storage properties and cycling stabilities. Additionally, the SIBs that were built functioned well in the −20 to −40 °C temperature range, opening up the possibility of using them for energy storage in harsh environments. S. Huang et al. constructed a structure by embedding FePS_3_ into graphitized porous N-doped carbon, which caused the abundant graphitized pores to accelerate the diffusion of Na^+^, and improved conductivity by constructing a conductive network of graphitized pore walls, as shown in Figure 6a–h [99]. The full-cell and half-cell matching NVPO@C also showed excellent performance under low temperatures and high loads. This work provides an important reference for the development of high-rate and -capacity anode materials and their low-temperature practicabilities.

### 3.2. Structural Engineering of Carbon-Based Anodes for Low-Temperature LIBs

The structural regulation of carbon-based materials is a promising technique for enhancing the low-temperature performances of LIBs and controlling electrons on the surface of carbon atoms. The internal structure of carbon-based materials can be controlled in the following six ways: improving electrolyte penetration of the surface area; rapid ion transport with short solid ion diffusion length; enhancing lithium storage electrochemical activity; improving the cycle stability of lithium-ion batteries; shortening the long diffusion path of lithium ions in graphite; and inhibiting the influence of lithium dendrites on the low-temperature performance of lithium ions. Thus, the low-temperature chemical performance of LIBs can be improved.

**Figure 7:**
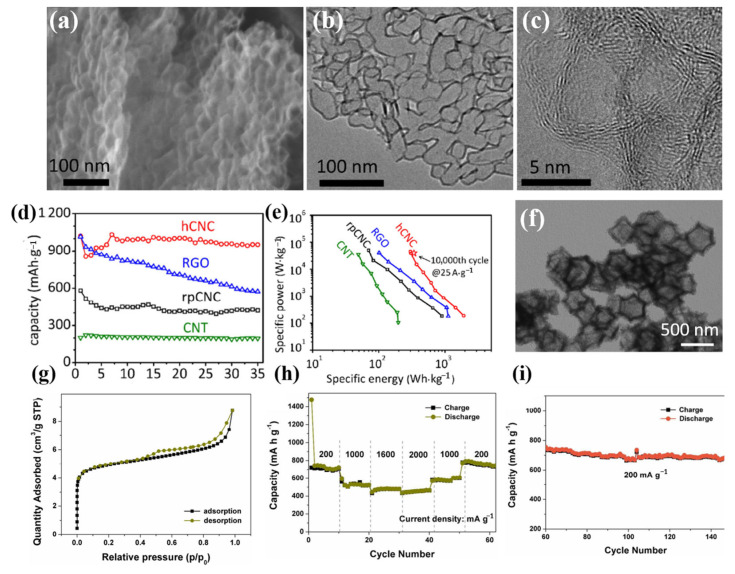
(**a**): SEM image. (**b**): TEM image. (**c**): High-resolution TEM image of the nanocages in (**b**) with well-graphitized layers in the shell. (**d**): The reversible capacities at the rate of 0.1 A g^−1^. (**e**): Ragone plot for the hCNC-based cell with lithium metal as the counter/reference electrode. The red pentagram represents the specific energy (339 Wh·kg_electrode_^−1^) and specific power (37 kW·kg_electrode_^−1^) for the hCNC after 10,000 cycles at the current density of 25 A·g^−1^. Comparison of Ragone plots for the hCNC and the rpCNC, CNT as well as RGO-based cells. (**a**–**e**) Reproduced with permission from [100]. (**f**): Low-magnification TEM image. (**g**): Nitrogen adsorption-desorption isotherms. (**h**): Rate performance at different current densities between 0.01 and 3.0 V. (**i**): Continued cycling performance of the porous Co-Zn/N-C polyhedral nanocage electrode (the same cell as for (**h**)) over 80 cycles at 0.2 A g^−1^. (**f**–**i**) Reproduced with permission from [101].

Carbon nanomaterials represent promising high-speed anodes for lithium-ion and sodium-ion batteries due to their plentiful carbon nanostructures, which enable electrolyte penetration with a high surface area and rapid ion transport with short solid-state ion diffusion lengths, and which can prevent bottlenecks caused by significant volume changes during ion intercalation/delamination. Ion diffusion, electron transport, and structural stability can all be effectively enhanced by using the three-dimensional layered structure of carbon nanomaterials. In this regard, Hu’s group prepared hCNC at 800 °C using the in situ magnesium oxide template method [100]. Its distinctive porous network structure is particularly advantageous for electron conduction, ion diffusion, structural stability, and electrolyte penetration, resulting in a high rate ability and long cycle life. After ten cycles, the matching electrode for lithium storage had a steady reversible capacity of 970 mAh g^−1^ at 0.1 A g^−1^ (Figure 7a–e). Interconnected hollow carbon nanocages with three-dimensional layered structures are visible in SEM and TEM pictures, and this is advantageous for the (de)insertion of Li ions. Additionally, Huang et al. created porous bimetallic Co/Zn-embedded N-doped carbon (Co-Zn/N-C) polyhedral nanocages by annealing the ZIF-8@ZIF-67 precursor in an argon atmosphere at 800 °C to address the issue of the low specific capacity (372 mAh g^−1^) of the graphite anode material in LIBs [101]. As shown in Figure 7f–i, the Co-Zn/N-C has a large specific surface area of 349.12 m^2^ g^−1^ and many micropores and mesoporous pores because nanoparticles are evenly distributed throughout the carbon matrix. The influence of volume change is reduced, and the electrical conductivity of the entire electrode is maintained. The lithium storage electrochemical activity is increased thanks to the distinctive porous hollow structure of Zn and Co and the synergistic effect of N doping. The initial discharge capacity of 809 mAh g^−1^ and capacity retention capacity after 400 cycles at a current density of 0.2 A g^−1^ of the porous Co-Zn/N-C polyhedral nanocages-based electrodes are beneficial for enhancing the low-temperature chemical performance of LIBs, and merit further research.

**Figure 8:**
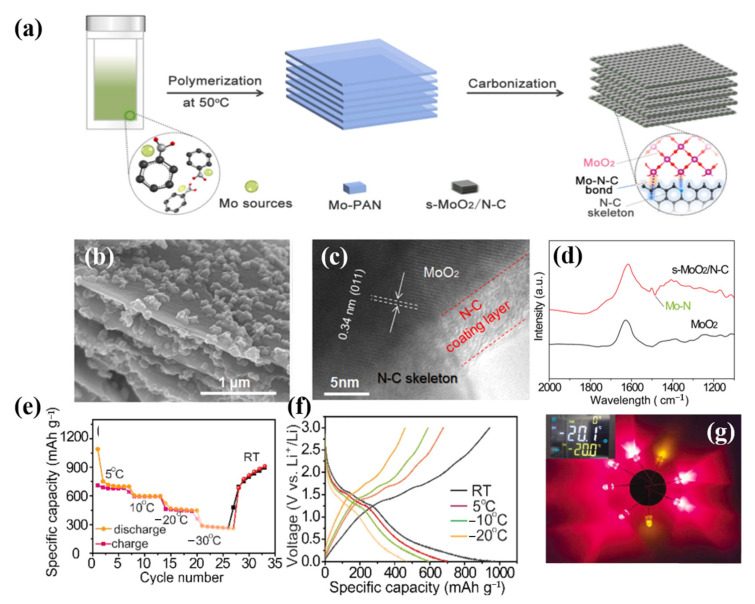
(**a**): Schematic illustration of the synthesis of s-MoO_2_/N-C with strong Mo-N-C chemical bonding interactions through a self-template method. (**b**): SEM images and their corresponding EDS elemental mappings of the prepared s-MoO_2_/N-C composite. (**c**): HRTEM images of s-MoO_2_/N-C. (**d**): FT-IR spectra of s-MoO_2_/N-C and commercial MoO_2_, respectively. (**e**): Capacity retention of s-MoO_2_/N-C at the temperature range of 5 °C to −30 °C. (**f**): Galvanostatic discharge/charge profiles (**g**): The photograph shows that the battery using the s-MoO_2_/N-C electrode can light up eight LED bulbs. (**a**–**g**) Reproduced with permission from [103].

Due to the advantages of small size and large specific surface area, MoO_2_ nanoparticles have far better charge transfer and storage performance than other materials, and are a good choice for LIBs. The inserted carbon-based substrate can simultaneously operate as a strong skeleton and an efficient conducting network to dampen the volume variations of MoO_2_ nanoparticles and increase their cyclic stability. MoO_2_ is therefore considered a potential electrode material for lithium-ion storage. However, in LIBs, MoO_2_ exhibits delayed kinetics and significant volume expansion/contraction. A workable method to enhance the electrochemical performance of MoO_2_-based materials has been thought of as the reasonable production of MoO_2_ nanoparticles and highly conductive carbon decorative materials, n-MoO_2_@C. In 2015, MoO_2_/C appeared for the first time [102]. The three-dimensional porous MoO_2_@C/graphene multistage structure of the composite material demonstrated remarkable electrochemical capabilities, including high capacity, long cycle life, and a steady, high rate. In 2022, Li et al. used a self-made template method to design and synthesize MoO_2_ and N-doped graded carbon nanoplates (s-MoO_2_/N-C) with strong surface bonding through an interface Mo-N-C bond, which demonstrated excellent lithium storage performance at low temperature, providing guidance for enhancing the carbon negative electrode for the low-temperature performance of LIBs (Figure 8) [103].

**Figure 9:**
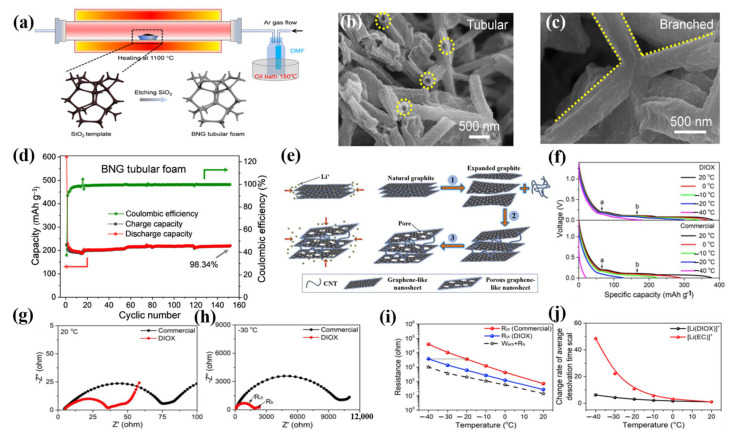
(**a**): Schematic diagram of the preparation of the BNG tubular foam using the self-sacrifice template CVD method. (**b**,**c**): SEM images of BNG tubular foam. (**d**): Cyclic performance of BNG tubular foam at a current density of 0.1 C. (**a**–**d**) Reproduced with permission from [89]. (**e**): Schematic illustration of the Li^+^ insertion process for natural graphite and porous graphite nanosheet (PGN). (**f**): Voltage vs. Li^+^ intercalation capacity over various temperatures at the 0.1 C rate in different electrolytes. (**g**,**h**): Nyquist plots of a PGN/CNT electrode measured at (**g**) 20 °C and (**h**) −30 °C in different electrolytes. (**i**): Difference of temperature-dependent resistance in different electrolytes. (**j**): Relative change rate of an average desolvation time scale of different solvated Li^+^ at different temperatures. (**e**–**j**) Reproduced with permission from [49].

The low-temperature performance of LIBs can be greatly enhanced by optimizing the relatively inert carbon network into an active material for lithium-ion storage at low temperatures [104]. For this, F. Lu et al. prepared branched N-doped graphitic tubular foam (BNG) as an anode material for high-performance LIBs at low temperatures using the self-fracture template CVD method, ensuring that the heterocompound nitrogen introduced was precisely distributed throughout the frame, increasing the spacing between planes, and enabling electronic optimization (Figure 9a–d) [89]. Since the design is dominated by pyridine/pyrrole defect C-N moieties and has curved knots and enlarged plane branches throughout the BNG tubular foam, LIBs are able to reach a reasonable capacity of 222.5 mAh g^−1^ at −10 °C. By achieving the anticipated cycle longevity and Coulombic efficiency, high-performance lithium storage at low temperatures can now be designed and optimized in greater detail. To further enhance the rate performance of lithium ions between the electrode and electrolyte interface and therefore enhance the low-temperature performance of LIBs, it is also appropriate to minimize the long diffusion path of lithium ions in graphite. Xu et al. synthesized nanosheets (PGN) and CNT using a novel carbon composite material composed of porous graphite [49]. CNTs can prevent graphite from reaccumulating and through-holes on porous graphite nanosheets can efficiently shorten the diffusion path. In PG/CNT composites, the predominant mesopore and few micropores encourage the rapid transmission of lithium ions, which allows LIBs to have strong rates and low-temperature performances (Figure 9e–j).

Despite significant progress, significant challenges remain in utilizing carbon-based materials for low-temperature LIBs, and the primary focus should be directed towards the following areas: (1): due to the significant influence of hierarchical porous structure on mass transport during energy storage and conversion, it is crucial to determine its role through theoretical modelling; (2) developing an electrochemical reaction mechanism; (3) how to adapt battery performance to extreme temperatures; (4) how to use first principles to calculate the electronic structure of carbon-based materials’ surfaces/interfaces and establish a mass transfer model, so as to find a way to reduce the energy barrier of Li^+^ migration.

## 4. Conclusions

With the increased demand for vehicles and energy storage powered by LIBs, there is a need for the study of anode materials with high capacities, long cycle lives, and wide temperature ranges. In particular, ways to overcome adverse factors such as slow dynamics at low temperatures are particularly critical for adaptation to a wider range of requirements. Carbon-based materials possess stable structures, excellent cycle stabilities and rate performances, and good lithium-ion diffusion abilities at low temperatures. The excellent low-temperature performance of LIBs can be obtained through electronic modulation and structural regulation. In detail: (1) Manipulating the configurations and positions of doping in carbon-based materials is both challenging and intriguing. The integration of dopants with topological defects, such as pentagon rings and micropore edges, offers a plethora of possibilities for exploring novel applications of carbon-based nanomaterials. New synthesis methods, including organic synthesis, are predicted to enable the exclusive attainment of particular target configurations. By finely regulating the components on the interior and exterior surfaces, the carbon shells can be transformed into a Janus surface, thus achieving synergistic functions that enhance surface redox reactions or enable spatial separation of coupled reactions in low-temperature LIBs; (2) Efficient methods for regulating the porous structure of carbon materials must be further developed. In particular, more convenient and scalable approaches than the simple capillarity method are required to regulate the mesopores, which are essential for practical applications of high-volumetric energy-density LIBs. Additionally, the precise regulation of perforated micropores throughout the shells via appropriate reactions would be valuable, as this is closely linked to the molecular sieving/blocking effect in energy storage; (3) The use of carbon-based materials to encapsulate or support foreign active materials has shown significant benefits in energy applications. Therefore, it is strongly advised to create more composites of this type, given their potentials for exceptional performance. Efficient and controllable methods are necessary for encapsulating these materials, as complete encapsulation within the carbon materials is not always straightforward and can be time-consuming. In many instances, the amount of material that can be loaded is still considerably below the theoretical limit. However, there are still some problems related to the efficient application of carbon-based materials which require further research. For example, the first Coulombic efficiency is an important test index of cathode materials for LIBs, and the identification of a battery system with a large specific surface area and high first coulomb efficiency is a focus for further research. In this respect, it is more important to match the electrolytic liquid system with low reducibility in order to reduce or avoid the formation of SEI film and maximize the first Coulombic efficiency. Secondly, the ion diffusion mechanism at low temperature currently relies on research experience and more research is required. In the next step, in situ XRD and TEM should be examined, so as to further determine the lithium storage mechanism using different structures.

## Figures and Tables

**Table 1:** Modified carbon-based materials for low-temperature LIBs.

Carbon-Based Materials	Test Temperature	Capacity	Current Density	Refs.
**PGN/CNT**	−40 °C	300 mAh g^−1^	20 C	Xu et al. [82]
**NiO@C-N NSs**	−40 °C	428 mAh g^−1^	0.05 A g^−1^	Bai et al. [83]
**MnO@Graphite**	−25 °C	295 mAh g^−1^	0.1 A g^−1^	Tian et al. [84]
**LTO/Ag/CNT**	−60 °C	140 mAh g^−1^	0.2 C	Hu et al. [85]
**TiO_2_(B)/Graphene**	−30 °C	240 mAh g^−1^	0.1 A g^−1^	Zhang et al. [86]
**Ti_3_C_2_Tx(O)**	−20 °C	88 mAh g^−1^	1 A g^−1^	Wang et al. [87]
**SFC**	−10 °C	76 mAh g^−1^	0.1 A g^−1^	Wang et al. [88]
**BNG**	−10 °C	223 mAh g^−1^	0.2 C	Lu et al. [89]
**N, S** **−Mo_2_C/C-ACF**	5 °C	405 mAh g^−1^	5 C	Li et al. [90]
**G/HTO**	−20 °C	211 mAh g^−1^	0.1 A g^−1^	Li et al. [91]

MnO nanoparticles anchored on graphite (MnO@Graphite); etching Ti_3_SiC_2_ in an atmosphere containing SF_6_(SFC); BNG tubular foam obtained by the chemical vapor deposition CVD method; Mo_2_C particles are embedded in the nitrogen-containing carbon framework (N,S−Mo_2_C/C-ACF); N-doped TiO_2_/TiN/graphene (G/HTO).

## Data Availability

Data is available on request.
